# Inability to Ventilate after Tube Exchange Postoperative to Pneumonectomy

**DOI:** 10.1155/2012/801093

**Published:** 2012-04-05

**Authors:** S. E. Verstraeten, A. H. M. van Straten, H. H. M. Korsten, E. W. G. Weber, P. L. M. L. Wielders, E. Berreklouw

**Affiliations:** ^1^Department of Cardiothoracic Surgery, Catharina Hospital Eindhoven, Michelangelolaan 2, 5623 EJ Eindhoven, The Netherlands; ^2^Department of Anesthesiology, Catharina Hospital Eindhoven, Michelangelolaan 2, 5623 EJ Eindhoven, The Netherlands; ^3^Department of Pulmonary Disease, Catharina Hospital Eindhoven, Michelangelolaan 2, 5623 EJ Eindhoven, The Netherlands

## Abstract

We report a case of inability to ventilate a patient after completion of pneumonectomy, due to migrated tumor tissue to the contralateral side. This represents an unusual complication with a high mortality rate. We have managed to find the cause in time and were able to remove the obstructive tissue using bronchoscopy.

## 1. Introduction

A pneumonectomy has sometimes to be performed for the management of a bronchus carcinoma [1, 2]. This procedure effects the anatomy of the chest dramatically and may lead to many complications. We present a case of pneumonectomy with a rare complication directly postoperatively. It appeared to be impossible to ventilate the patient properly after changing the double lumen tube to a regular single lumen tube. 

## 2. Case Presentation

**Figure 1:**
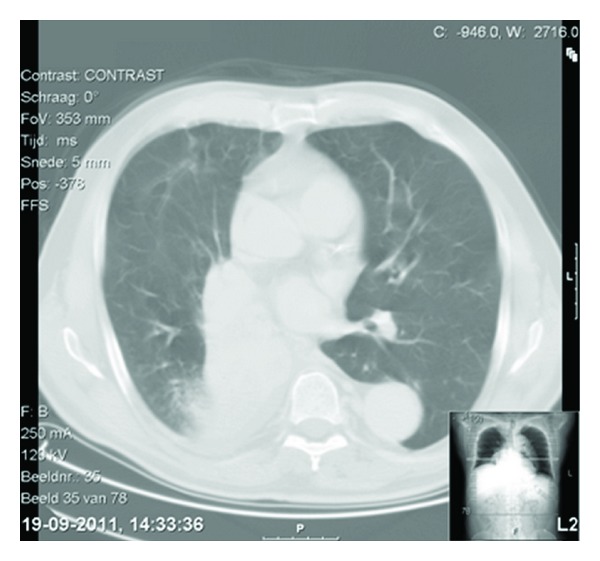
CT scan.

A 70-year-old Caucasian male patient was referred to the pulmonologist by his general practitioner because of an abnormal X-ray of the chest. Computed tomographic (CT) scanning showed a large central tumor near the right main bronchus (Figure 1). Bronchoscopy showed a complete obstruction of the bronchus intermedius by tumor tissue, almost two centimeters from the main carina. The diagnosis was confirmed after performing a puncture, which showed a non-small-cell bronchus carcinoma.

A total right pneumonectomy was performed with extensive lymph node sampling. During the procedure, the patient tolerated single lung ventilation moderately. Bilateral lung ventilation was sometimes needed to maintain adequate pulse oxygen saturation levels. Despite that, the patient was hemodynamically stable during the entire procedure.

After completion of the procedure, the double lumen tube was changed into a single lumen tube. Thereafter, it appeared to be impossible to ventilate the patient properly. Manual ventilation was impossible, even with extremely high pressures. Pulse oxygen saturation dropped to beneath thirty percent, while capnography did not show any signs of returning carbon dioxide. 

Though the single lumen tube was placed properly, it was replaced by another single lumen tube, without any respiratory improvement. To exclude the possibility of a pneumothorax, the perioperative-placed chest tube was disconnected and a second chest tube was inserted to the left pleura, but these actions did not resolve the problem. Meanwhile, the patient became further unstable: pulse oxygen saturation remained below thirty percent, arterial systolic blood pressure decreased to beneath 50 mmHg, while heart frequency rose to over 140 beats per minute. Therefore, epinephrine was administered to correct the hemodynamic condition. Bronchodilators and hydrocortisone did not appear to be beneficial. 

**Figure 2:**
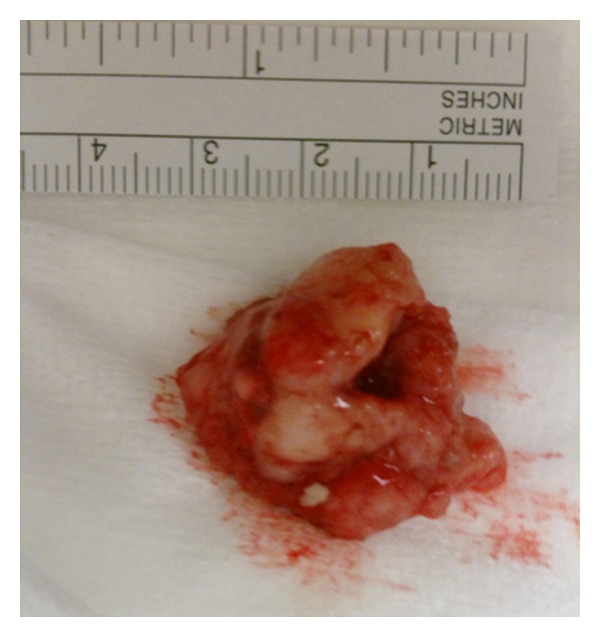
Tumorous tissue causing the bronchial obstruction.

However, ventilation seemed to improve a little by rotating the patients head to the right, which suspects an embolus. Fibreoptic bronchoscopy showed obstruction of the left main bronchus with a chunk of tissue (Figure 2). A rigid bronchoscopy was performed, and the obstructing tissue was removed using a gripping device. Directly after this maneuver, the patient could be ventilated properly again. Oxygen saturation levels and arterial blood pressure increased rapidly to normal levels.

Histopathologic examination revealed an adenocarcinoma located in the right middle lobe without invasion of the pleura. The diameter of the tumor was five centimeters. Of the nineteen resected lymph nodes, two contained metastases. The chunk of tissue held responsible for the ventilation problems was indeed tumor tissue.

The postoperative course was uneventful. No neurological damage was detected. The patient was discharged from the hospital seven days after the procedure. 

The position of the left double lumen tube (Carlens) was preoperatively checked using flexible bronchoscopy. At that time, no obstructions were seen in the left main bronchus. During surgery, a piece of tumor tissue must have gotten detached from the main tumor. Since the tube was situated properly and the cuff was inflated, obstruction of the left main bronchus was impossible. However, after repositioning the patient in supine position and removing the double lumen tube, the left main bronchus was exposed. Hence, the tumor tissue had the opportunity to migrate to the contralateral side. When ventilation was resumed, the positive air flow stimulated the tissue to move distally and obstruct the left main bronchus.

## 3. Discussion

Endotracheal bronchus obstruction after pneumonectomy is a rare complication that comes with great mortality, [3–8]. It was first described in 1966 by Fox and colleagues and thereafter a few more cases were published [9]. 

Obstruction can occur, like in our case, after surgery, but it may also happen during the procedure itself. Logically, a single lumen tube increases the chance of intraoperative obstruction. Nevertheless, using a double lumen tube may not prevent the complication but may only cause a delay. 

If such a problem, a difficulty to ventilate after completion of a pneumonectomy, occurs, the first step in the management is to check the position of the new single lumen tube using a laryngoscope. Auscultation is an easy act to check whether the remaining lung is ventilated, which can be confirmed by capnography. When the tube is positioned properly, one should check whether the thoracic drain is functioning. If there is air leakage through the bronchial stump, it may cause a tension pneumothorax. To treat a possible tension pneumothorax contralaterally, a drain could be inserted. The final step in the management is the use of a fibreoptic bronchoscope to exclude any mechanical problems. 

Prevention of such a complication can be (partially) achieved by fibreoptic bronchoscopy before rigid bronchoscopy during the ventilation tube switch. In this way, detached tumor fragments may be detected in time. When fragments are present, these can be removed using forceps. 

Some authors suggested inspection of the carina through the bronchial stump, but this is not an option with the present techniques of automatic stapling devices.
